# Comparison of three distinct surgical clothing systems for protection from air-borne bacteria: A prospective observational study

**DOI:** 10.1186/1754-9493-6-23

**Published:** 2012-10-15

**Authors:** Ann Tammelin, Bengt Ljungqvist, Berit Reinmüller

**Affiliations:** 1 Department of Medicine, Solna (MedS), Unit of Infectious Diseases, Karolinska Institutet, Stockholm, Sweden; 2 Department of Energy and Environment, Division of Building Services Engineering, Chalmers University of Technology, Göteborg, Sweden; 3 Department of Infection Control and Hospital Hygiene, Stockholm County Council, Södersjukhuset, hiss S, plan −1, Stockholm, SE-118 83, Sweden

**Keywords:** Orthopaedic surgery, Protective clothing, Ventilation

## Abstract

**Background:**

To prevent surgical site infection it is desirable to keep bacterial counts low in the operating room air during orthopaedic surgery, especially prosthetic surgery. As the air-borne bacteria are mainly derived from the skin flora of the personnel present in the operating room a reduction could be achieved by using a clothing system for staff made from a material fulfilling the requirements in the standard EN 13795. The aim of this study was to compare the protective capacity between three clothing systems made of different materials – one mixed cotton/polyester and two polyesters - which all had passed the tests according to EN 13795.

**Methods:**

Measuring of CFU/m^3^ air was performed during 21 orthopaedic procedures performed in four operating rooms with turbulent, mixing ventilation with air flows of 755 – 1,050 L/s. All staff in the operating room wore clothes made from the same material during each surgical procedure.

**Results:**

The source strength (mean value of CFU emitted from one person per second) calculated for the three garments were 4.1, 2.4 and 0.6 respectively. In an operating room with an air flow of 755 L/s both clothing systems made of polyester reduced the amount of CFU/m^3^ significantly compared to the clothing system made from mixed material. In an operating room with air intake of 1,050 L/s a significant reduction was only achieved with the polyester that had the lowest source strength.

**Conclusions:**

Polyester has a better protective capacity than cotton/polyester. There is need for more discriminating tests of the protective efficacy of textile materials intended to use for operating garment.

## Background

Since the 1970s, it is a generally accepted view that the amount of bacteria in the operating room air should be as low as possible in orthopaedic prosthetic surgery to prevent postoperative infections related to the implant. The air-borne bacteria that reach the surgical site are mainly staphylococci derived from the skin flora of the personnel present in the operating room. This was presented in historic landmark articles by Charnley and Lidwell [1,2] and has later been confirmed by other authors [3,4]. In order to achieve low bacteria levels it is possible to use ventilation to dilute and/or swipe away the bacteria-carrying particles in the air [5]. One can also use a clothing system made of a material that is so dense that the bacteria-carrying skin scales which continuously come loose from the outer skin layer does not reach the air in the room [6,7].

Since the year 2009 there is a European standard, EN 13795, which describes the requirements imposed on the material density for a dress to be classified as a so-called Clean Air Suite [8]. In Swedish orthopaedic surgery the most commonly used dress for operating room personnel is made of cotton/polyester. This material meets the standard requirements when it is brand new. The clothes are intended for multiple use and therefore undergo a large number of washing processes during their lifetime. The repeated washing could lead to a change of the material properties so that it becomes more permeable to bacteria-carrying particles. In some Swedish hospitals the operating room staff use dresses made of polyester, and there are indications that this garment would have a better protective effect than the standard clothing system made from the mixed material [5].

Based on the measured total amount of Colony Forming Units (CFU) per cubic meter (m^3^) air, the air flow and the number of people in the operating room it is possible to calculate the protective efficacy of a surgical clothing system in terms of source strength (mean value of CFU emitted from one person per second), which makes it possible to compare the protective capacity of clothing made from different materials [9].

In this study we wanted to investigate whether there was any difference in protective efficacy between clothing systems made of polyester (two different materials) and a mixed material (cotton/polyester), all of which meet the requirements of standard EN 13795. We also wanted to investigate if there was reason to believe that the protective effect may be impaired in garments of mixed material which had been washed repeatedly.

## Methods

### Setting

The study was conducted at South Hospital (Södersjukhuset), Stockholm, in 2010. South Hospital is an emergency hospital with approximately 650 beds where about 6,000 orthopaedic surgical procedures are performed each year.

A total of 21 orthopaedic surgical procedures performed in the operating rooms 1, 2, 3 and 5 were included in the study. The operating theatres had turbulent, mixing ventilation with air flow (air intake) at 996, 965, 1,050 and 755 L/s respectively. The selection of surgical procedures was haphazard and made mainly by the availability of personnel who could perform the sample collection.

**Figure 1:**
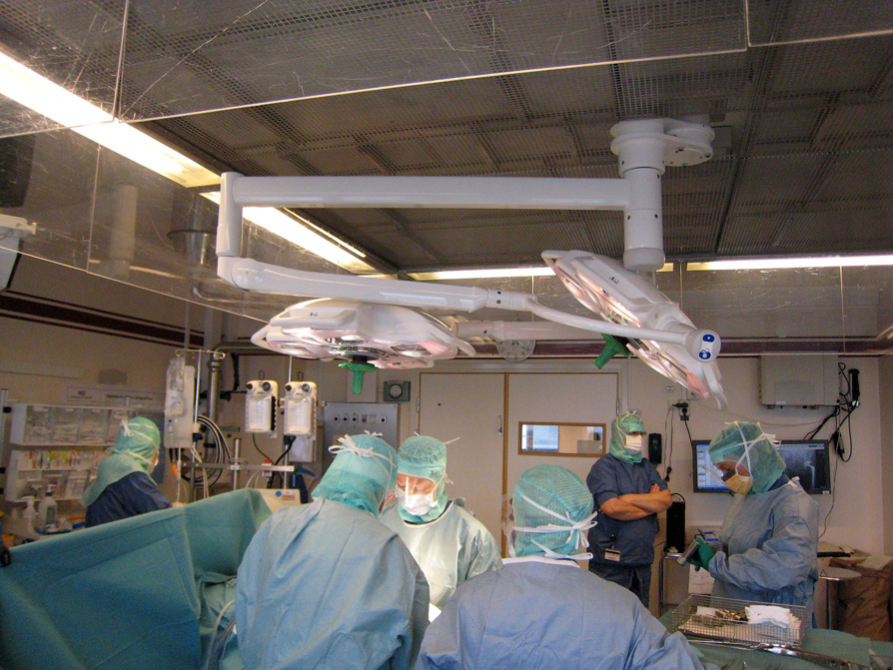
Operating staff dressed in clothes made from mixed material.

All present staff (5 – 9 persons) in the operating room wore clothes made from the same material during each surgical procedure. All dresses were of the same design i.e. the trousers had cuffs at the leg and the short-sleeved shirts had cuffs at the arm, bottom and neckline (Figure 1).

### Clothing systems

The three clothing systems studied were made from the following fabrics:

Mixed material (Mertex P-3477^®^, Mercan AB) consisting of 69% cotton, 30% polyester, and 1% carbon fibre. Weight 150 gram per square meter.

Polyester material (Mertex HK-1069KS^®^, Mercan AB) consisting of 99% polyester and 1% carbon fibre. Weight 100 gram per square meter.

Polyester material (Selguard 4^®^, 807TK-310, Martinson Konfektion AB) consisting of 99% polyester and 1% carbon fibre. Weight 120 gram per square meter.

In operating room 1 we made a comparison between the routinely used dresses that had been washed for several times (up to a maximum of 100 washing processes) picked from the shelf (two operations) and brand new dresses (two operations), all made from the mixed material. In operating room 2 air-samples were taken during three operations with all staff using washed dresses made from the mixed material.

In operating rooms 3 and 5 sampling was performed during six surgical procedures where staff wore dresses made from mixed material and eight surgical procedures where they used dresses made from polyester.

### Sample collection and microbiological analysis

**Figure 2:**
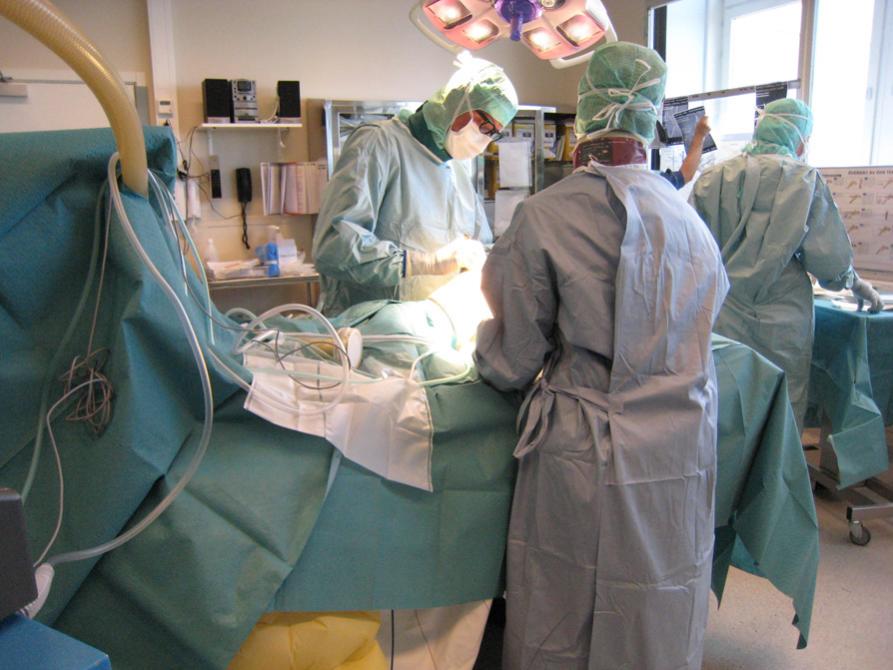
Position of holder for sterile gelatine filter.

Measurements of CFU/m^3^ air were carried out by personnel from the laboratory for clinical microbiology at the Karolinska University Hospital in Huddinge. Air sampling was made with a Sartorius MD8 air sampler with a flow of 100 L/min for periods of 10 minutes (thus sampled air volume of 1.0 m^3^). Air was sucked over a sterile gelatine filter placed as near the surgical wound as possible (approximately 20 – 50 cm). (Figure 2) At each operation air was sucked for four-six ten-minute periods. Each gelatine filter was placed on a sterile blood agar plate that was incubated at 35°C for two days. The number of bacterial colonies on the plate was then counted and expressed as CFU/m^3^ air. From the four-six samples a mean value of CFU/m^3^ air was calculated for each operation.

### Data analysis

Mann–Whitney *U*-test, two-sided, was used when comparing means of CFU/m^3^ air from operations when washed and brand new clothes made from mixed material were used.

Mann–Whitney *U*-test, one-sided, was used when comparing means of CFU/m^3^ air from operations when clothes made from mixed material and polyester were used, with the hypothesis that polyester would give lower counts of CFU/m^3^ air.

Mann–Whitney *U*-test, one-sided, was used when comparing means of source strength calculated from operations when clothes made from mixed material and polyester were used, with the hypothesis that polyester would result in lower source strength. The same test was used when comparing source strength for the two polyesters, with the hypothesis that Selguard 4^®^, 807TK-310 would have a lower source strength.

P-values ≤ 0.05 were considered significant.

### Ethical approval

This study has not been subject to judgement by an ethics committee as it was regarded as a quality project.

## Results

### Washed and brand new dresses made from mixed material (cotton/polyester)

In operating room 1 we found no significant differences in the number of CFU/m^3^ air between the two operations in which staff wore washed clothes (mean 29.3) and the two when brand new ones were worn (mean 24.3), all made from mixed material (Table 1).

**Table 1:** **Mean values of CFU/m**^**3 **^**air during four operations in operating room 1 when all persons present were dressed in a surgical clothing system of mixed material (69% cotton, 30% polyester, 1% carbon fibre) which was either washed repeatedly or brand new**

**Type of surgery**	**Clothing**	**Number of persons present**	**CFU/m3 air mean (min-max)**
Hip fracture	Mixed material, washed	7	38.3 (17–55)
Hip fracture	Mixed material, washed	8	20.2 (9–32)
Lower leg fracture	Mixed material, brand new	7	16.7 (13–24)
Lower leg fracture	Mixed material, brand new	7	31.8 (8–50)

When comparing all operations performed with staff dressed in washed and brand new clothes made from mixed material irrespective of operating room the mean values (CFU/m^3^) were 27.7 and 34.5 respectively. The difference was not significant (Tables 1, 2, 3 and 4).

**Table 2:** **Mean values of CFU/m**^**3 **^**air during three operations in operating room 2 when all persons present were dressed in a surgical clothing system of mixed material (69% cotton, 30% polyester, 1% carbon fibre) which was washed repeatedly**

**Type of surgery**	**Clothing**	**Number of persons present**	**CFU/m3 airmean (min-max)**
Wrist	Mixed material, washed	5	41.3 (9–65)
Hamstring muscle	Mixed material, washed	7	24.0 (17–38)
Shoulder	Mixed material, washed	7	31.0 (11–45)

**Table 3:** **Mean values of CFU/m**^**3 **^**air during seven operations in operating room 3 when all persons present were dressed in surgical clothing systems of either mixed material (69% cotton, 30% polyester, 1% carbon fibre) or polyester (99% polyester, 1% carbon fibre) of two kinds**

**Type of surgery**	**Clothing**	**Number of persons present**	**CFU/m3 air mean (min-max)**
Back, infected	Mixed material, washed	7	9.8 (1–20)
Ankle	Mixed material, washed	7	34.7 (20–49)
Shoulder, clavicle	Mixed material, washed	7	22.2 (14–37)
Ankle	Polyester HK-1069KS	8	12.7 (7–16)
Hip replacement	Polyester HK-1069KS	6	27.2 (10–40)
Hip replacement	Polyester 807TK-310	7	3.2 (1–6)
Ankle fracture	Polyester 807TK-310	7	5.3 (2–12)

**Table 4:** **Mean values of CFU/m**^**3 **^**air during seven operations in operating room 5 when all persons present were dressed in surgical clothing systems of either mixed material (69% cotton, 30% polyester, 1% carbon fibre) or polyester (99% polyester, 1% carbon fibre) of two kinds**

**Type of surgery**	**Clothing**	**Number of persons present**	**CFU/m3 air mean (min-max)**
Hip	Mixed material, brand new	7	46.5 (40–58)
Knee	Mixed material, brand new	8	49.0 (37–88)
Knee	Mixed material, brand new	6	28.6 (12–50)
Knee replacement	Polyester HK-1069KS	Not noted	7.3 (5–10)*
Knee	Polyester HK-1069KS	7	7.0 (1–13)
Knee	Polyester 807TK-310	8	5.2 (2–8)
Knee	Polyester 807TK-310	7	4.3 (2–9)

* Value not used in calculation of source strength (Table 4).

### Mixed material (cotton/polyester) and polyester

When we compared the results obtained with clothes made from mixed material (washed and brand new) and the two polyesters (added results from both HK-1069KS and 807TK-310) in operating rooms 3 and 5 there was no significant difference between mixed material and polyester in operating room 3 but the difference was significant in room 5 (Tables 3 and 4).

When we added all results (from room 3 and 5) obtained with the clothing system made from mixed material and compared with the added results (from room 3 and 5) obtained with each of the clothing systems made from polyester (HK-1069KS and 807TK-310 respectively) the difference in CFU/m^3^ was significantly lower in favour of each of the polyesters (Tables 3 and 4).

Due to few operations included it was not possible to tell if the difference in number of CFU/m^3^ was significant when making the same comparison between mixed material and each of the polyesters for the two operating rooms 3 and 5 separately.

### Source strength

Source strength was calculated for the different clothing systems used (washed and brand new mixed material, polyester HK-1069KS and polyester 807TK-310). The difference between new and washed clothes made from mixed material was not significant, neither was the difference between clothes made from mixed material and polyester HK-1069KS or between the two polyesters. The difference between mixed material and polyester 807TK-310 was significant. (Table 5)

**Table 5:** Source strength (mean value of CFU emitted from one person per second) calculated for each clothing system during each operation

**Clothing system**	**Source strength (CFU/s) mean (min – max)**
Mixed material, washed	4.2 (1.5 – 8.0)
Mixed material, brand new	4.0 (2.4 – 5.0)
Polyester HK-1069KS	2.4 (0.8 – 4.8)
Polyester 807TK-310	0.6 (0.5 – 0.8)

## Discussion

According to the company (Textilia AB) that supplies clothing to the hospital the density of Clean Air Suits made from mixed material has been tested after 120 washing processes and was then found to meet the standard's requirements. The supplier warrants that each piece of garment is eliminated after a maximum of 100 washing processes. Our results support that there is no significant difference between the washed clothes routinely used during orthopaedic surgery and the brand new clothes, both made from mixed material, with respect to their ability to protect the patient from air-borne bacteria coming from the personnel.

Already in 1990 Whyte et al. showed that surgical clothing made of polyester was superior to cotton clothing with respect to reduction of air-borne bacteria in an operating room with conventional, turbulent, mixing ventilation [10]. The same was shown by Verkkala et al. in 1998 [11]. In spite of that, cotton has not been replaced by polyester but by the mixed material (cotton/polyester) for routine use in Swedish orthopaedic surgery. This might partly be explained by promising results from studies with surgical clothing made from mixed material in operating theatres supplied with partial unidirectional air flow [12]. As most operations for hip and knee replacement in Sweden still are performed in operating theatres with mixing, turbulent air flow it seems important to investigate whether it is possible to improve air quality in this kind of operating rooms by using polyester garment.

Our overall results show that a dress in polyester is able to reduce bacterial counts in the operating room air to a significantly greater extent than the commonly used dress in mixed material even though the material used for garment of each type meets the requirements for Clean Air Suite due to the standard EN 13795. The test methods specified in the standard thus seems suitable to outrange materials that are totally inappropriate for use in surgical clothing systems intended to be medical devices, i.e. clothes that should protect the patient from air-borne bacteria. For materials passing the tests it is though impossible to distinguish between those having source strengths of below 1 and around 4 CFU/s respectively. Under real conditions in the operating room this difference in source strength leads to differences in CFU/m^3^ air ranging from below 5 to around 50.

The operating rooms 1, 2 and 3 all had an air intake of approximately 1,000 L/s whereas room 5 had an intake of only 755 L/s. The lower air-flow in room 5 has probably resulted in the higher mean value of 41.4 CFU/m^3^ (min 12, max 88) compared to the mean value of 27.0 CFU/m^3^ (min 1, max 65) in the rooms 1, 2 and 3 when adding results from all operations performed with dresses in the mixed material. This supports the theory that a higher air intake helps to keep low levels of bacteria in the air by dilution. The difference in air intake affected the final results as shown below.

Our results indicate that with a conventional, turbulent, mixing ventilation with an air flow of about 1,000 L/s (15–20 air changes per hour) it is not possible to expect a microbiological air quality with the desired level of <10 CFU/m^3^ when using clothes in mixed material giving a mean source strength of about 4 CFU/s, although the material meets the requirements in EN 13795.

Of the two polyester fabrics tested one - Selguard 4^®^, 807TK-310 - showed to result in a significantly lower source strength than the mixed material. If the standard EN 13795 should be able to show such differences in protective efficacy it has to be completed with more sophisticated test methods. Today we have to rely on investigations in dispersal chambers or operating theatres to find them.

In this study the operating rooms 3 and 5 hade air flows of 1,050 and 755 L/s respectively. In room 5 we obtained a higher mean value of CFU/m^3^ than in room 3 when dresses in mixed material were used (41.4 versus 22.2 for all operations) and the reduction achieved by polyester garment was significant in room 5 but not in room 3. The result is not surprising. With a high baseline the air quality was improved also when results with the polyester Mertex HK-1069KS^®^ were included although this polyester had a lower protective capacity. In room 3 with a lower baseline the number of operations with dresses made from Selguard 4^®^, 807TK-310 unfortunately were too few to show a significant reduction. We are however convinced that this fabric with a mean source strength of 0.6 CFU/s has contributed to a mean value of 4.3 CFU/m^3^ for the two operations where it was used although the difference was not significant.

It is worth notice that the source strength for the clothing systems made from mixed material and from the polyester Mertex HK-1069KS^®^ varied substantially between operations whereas the variation was less for the polyester Selguard 4^®^, 807TK-310 (Table 5). When choosing a clothing system it is desirable that the user could expect the protective capacity to be stable.

In this study only a small number of surgical procedures with each clothing system were investigated and the results need to be confirmed by further studies. Another limitation could be that different kind of operations were studied as activity and performance differs which might lead to higher or lower levels of bacterial contamination of the air. It is however noteworthy that the CFU-levels could differ almost two-fold between operations of the same kind performed in the same operating room with the same number of staff present and using the same kind of clothing, which indicates that there are both low and high shedders of skin scales among the staff (Table 1).

## Conclusions

Clothing systems made of polyester has a better protective capacity than those made of cotton/polyester. There is need for more discriminating tests of the protective efficacy of textile materials intended to use for operating garment.

## Competing interests

None of the authors have any competing interests.

## Authors’ contributions

AT conceived of the study, and participated in its design and coordination and drafted the manuscript. BL participated in the design of the study and helped to draft the manuscript. BR participated in the design of the study and its coordination and participated in the sampling during operations and helped to draft the manuscript. All authors read and approved the final manuscript.

## References

[B1] Charnley J Postoperative infection after total hip replacement with special reference to air contamination in the operating room Clin Orthop Relat Res 1972 87 167 187 10.1097/00003086-197209000-00020 4562188

[B2] Lidwell OM Air, antibiotics and sepsis in replacement joints J Hosp Inf 1988 11 Suppl C 18 10.1016/0195-6701(88)90020-5 2899118

[B3] Whyte W Hambraeus A Laurell G Hoborn J The relative importance of the routes and sources of wound contamination during general surgery II. Airborne J Hosp Inf 1992 22 1 41 54 10.1016/0195-6701(92)90129-A 1358946

[B4] Edmiston CE Seabrook GR Cambria RA Brown KR Lewis BD Sommers JR Krepel CJ Wilson PJ Sinski S Towne JB Molecular epidemiology of microbial contamination in the operating room environment: Is there a risk for infection? Surgery 2005 138 4 573 579 10.1016/j.surg.2005.06.045 16269284

[B5] Nordenadler J Some observations on safety ventilation in operating rooms 2010 Royal Institute of Technology, Stockholm, Building Services Engineering: PhD thesis

[B6] Tammelin A Domicel P Hambraeus A Ståhle E Dispersal of methicillin-resistant Staphylococcus epidermidis by staff in an operating suite for thoracic and cardiovascular surgery: relation to skin carriage and clothing J Hosp Inf 2000 44 119 126 10.1053/jhin.1999.0665 10662562

[B7] Reinmüller B Ljungqvist B Evaluation of cleanroom garments in a dispersal chamber – some observations Eur J Parent Sci 2000 5 55 58

[B8] European Committee for Standardization, European Standard EN 13795, ICS 11.140 http://www.sis.se/hälso-och-sjukvård/sjukvårdstextilier-allmänt/ss-en-137952011.

[B9] Ljungqvist B Reinmüller B Nordenadler J Performance of clothing systems in the context of operating rooms: a question of patient safety Clean Air and Containment Review 2011 7 10 13

[B10] Whyte W Hamblen DL Kelly IG Hambraeus A Laurell G An investigation of occlusive polyester surgical clothing J Hosp Inf 1990 15 363 374 10.1016/0195-6701(90)90093-4 1972952

[B11] Verkkala K Eklund A Ojajärvi J Tiittanen L Hoborn J Mäkelä P The conventionally ventilated operating theatre and air contamination control during cardiac surgery – bacteriological and particulate matter control garment options for low level contamination Eur J Card-thor Surg 1998 14 206 210 10.1016/S1010-7940(98)00150-X 9755009

[B12] Tammelin A Hambraeus A Ståhle E Routes and sources of Staphylococcus aureus transmitted to the surgical wound during cardiothoracic surgery: Possibility of preventing wound contamination by use of special scrub suits Inf Cont Hosp Epid 2001 22 338 346 10.1086/501910 11519910

